# Validation of the Cantonese version of the Traditional Chinese Medicine (TCM) Body constitution Questionnaire in elderly people

**DOI:** 10.1186/s13020-023-00805-w

**Published:** 2023-10-11

**Authors:** Yiu Lin Wong, Jialing Zhang, Xingyao Wu, Suet Yee Wong, Zheng Wang, Linda L. D. Zhong, Zhaoxiang Bian

**Affiliations:** 1 School of Chinese Medicine, Hong Kong Baptist University, 3/F, Jockey Club School of Chinese Medicine Building, 7 Baptist University Road, Kowloon Tong, Hong Kong SAR, China; 2 Department of Social and Behavioural Sciences, City University of Hong Kong, Kowloon Tong, Hong Kong SAR, China; 3 Department of Applied Mathematics, The Hong Kong Polytechnic University, Hung Hom, Hong Kong SAR, China

**Keywords:** TCMECQ-C, Body constitution, Content validity, Factor analysis, Construct validity, Cronbach’s alpha, Intra-class correlation coefficients

## Abstract

**Background:**

The Traditional Chinese Medicine (TCM) Body Constitution Questionnaire (For Elderly People) (TCMECQ) is a patient-reported outcome questionnaire developed in Mandarin in 2013 to differentiate the body constitutions of the elderly aged 65 and above. Considering the cultural and linguistic differences between Mainland China and Hong Kong (HK) Special Administrative Region, the TCMECQ was translated into Cantonese following “back translation” policy and validated in proper process.

**Methods:**

Ten Chinese Medicine Practitioners (CMPs) and 30 senior citizens aged 65 or above were recruited to evaluate the first version of the Traditional Chinese Medicine Body Constitution Questionnaire (For Elderly People) (Cantonese version) (TCMECQ-C). Based on their comments, the second version was developed and discussed in the panel meeting to form the third version, validated the third version on 270 recruited seniors. Based on the validation results, a panel of 5 experts finalized the Questionnaire as the final version. The TCMECQ-C developers finalized the Questionnaire as the validated endorsed third version (i.e. final version).

**Results:**

The item-level content validity index of most items of the TCMECQ-C (First Version) were ranging from 0.80 to 1.00 in terms of clarity, relevance and appropriateness. Factor loadings of Qi-deficiency Constitution ranging from 0.37 to 0.71, Yang-deficiency Constitution ranging from 0.36 to 0.65, Yin-deficiency Constitution ranging from 0.36 to 0.65, and Stagnant Qi Constitution ranging from 0.68 to 0.82. The chi-squared degree-of-freedom ratio was 2.13 (928.63/436), Goodness-of-Fit Index (0.83), Adjusted Goodness-of-Fit Index (0.79), Normed Fit Index (0.66), Comparative Fit Index (0.78), Incremental Fit Index (0.78), Relative Fit Index (0.61) and Tucker–Lewis Index (0.75), and Root Mean Square Error of Approximation (0.07) and Standardized Root Mean Square Residual (0.07), implied acceptable Confirmatory Factor Analysis model fit of the overall scale. A Pearson correlation coefficient (r) showed the sufficient convergent validity for excessive subscales (Phlegm-dampness Constitution and Dampness-heat Constitution with r = 0.35, p < 0.01). Cronbach’s alpha coefficient ranged from 0.56 to 0.89, including Qi-deficiency Constitution (0.67), Yang-deficiency Constitution (0.84), Yin-deficiency Constitution (0.59), Stagnant Blood Constitution (0.56), Stagnant Qi Constitution (0.89), Inherited Special Constitution (0.76) and Balanced Constitution (0.73), indicating acceptable internal consistency for subscales. The intra-class correlation coefficients of the TCMECQ-C ranged from 0.70 to 0.87 (p < 0.001), indicating moderate to good test–retest reliability.

**Conclusion:**

TCMECQ-C is a valid and reliable questionnaire for assessing the body constitution in Cantonese elderly.

**Supplementary Information:**

The online version contains supplementary material available at 10.1186/s13020-023-00805-w.

## Introduction

In Traditional Chinese Medicine (TCM) theory, body constitution is an important concept for distinguishing individual human differences. One of the earliest medical texts in Ancient China, *the Yellow Emperor’s Book of Internal Medicine*, defines body constitution as “body quality nature or body build” [1]. It describes constitutions based on Yin-Yang theory, the five elements, body posture, functional characteristics, mental/emotional characteristics [1]. During the period of Eastern Han Dynasty, “Miracle Healer” Zhang Zhongjing classified individual human health based on the relationship between constitution and the incidence, nature, development, and prognosis of diseases [1]. In the late 1970s, Wang codified these general descriptions into nine specific fundamental body constitutions and developed the nine body constitution theory taking into account the internal and external factors of individuals, groups and environments [1, 2]. The fundamental principle can be described as “harmonization between soma and spirit” and is in line with TCM’s fundamental goal, of achieving harmony between human and nature [1, 2]. The theory guides therapy strategies, such as modification of lifestyle for long-term health and appropriate immediate measures to prevent evolution of a health deterioration [3, 4]. Accustomed to the innate and acquired endowments of the body constitution throughout the human life process, the identification of the constitution is a key step in TCM prevention therapy [1].

With the guidance of the nine body constitution theory, the Constitution in Chinese Medicine Questionnaire (CCMQ), targeting Mandarin-speaking population aged 15 years old and above [6], was developed by Wang and endorsed by the China Association of Chinese Medicine in 2009 [4]. Since then, the questionnaire has been applied to investigate the health conditions of various demographic groups in China [4]. Prior to the development of CCMQ, it was challenging for practitioners to accurately identify the patients’ body constitution in clinical practice, not to mention achieving interrater consistency between practitioners. Consequently, the valid and reliable CCMQ becomes utmost important to facilitate the standardization and accuracy in identifying the human body constitution. It also serves as an efficient and practical resource for large-scale observational research or community health prevention promotion [7].

The Japanese, Korean, English and Cantonese versions of CCMQ have been validated [8–12]. In 2013, Wong et al. found the Cantonese version to be valid and reliable for Hong Kong (HK) population [12]. However, in 2013, it was found that CCMQ is not entirely suitable for the elderly population due to considerations such as the cultural background of Chinese elderly and the structure of Chinese society, Wang et al. developed the Traditional Chinese Medicine Body Constitution Questionnaire (For Elderly People) (TCMECQ) based on CCMQ specifically for the elderly aged 65 and above in Beijing [13–15]. The TCMECQ comprising 33 questions, which is shorter than the 60 questions in CCMQ. The shortened questionnaire, questions tailored to elderly constitution characteristics, and easy-to-interpret scoring method promote the use of TCMECQ [5, 16]. The TCMECQ has paved the way for the standardization and accurate identification of body constitution for elderly. As the proportion of Cantonese-speaking elderly in HK is increasing [17], a Cantonese translation of TCMECQ is needed [12]. The objective of this study was to translate and validate the TCMECQ-C among the elderly aged 65 and above in HK.

## Material and methods

### Ethical approval

An instrument translation, evaluation and validation study were carried out from June 2019 to February 2020. The study instrument was approved by the Research Ethics Committee (REC) of Hong Kong Baptist University (HKBU) (Ref. No.: REC/18-19/0021). It was registered in ClinicalTrials.gov (ClinicalTrials.gov ID: NCT04491890) on July 26, 2020.

### Participants

The CMPs for content validity were recruited from the outpatient clinics of the School of Chinese Medicine (SCM) of HKBU; the participants of evaluation and validation were recruited from the outpatient clinics of the SCM of HKBU and the community started from July 2019 to February 2020. Eligible participants were aged 65 and above HK residents, capable of consenting and agreed to participate. The potential participants who have serious diseases including serious mental or behavioral disorders were excluded.

### Procedures

**Fig. 1:**
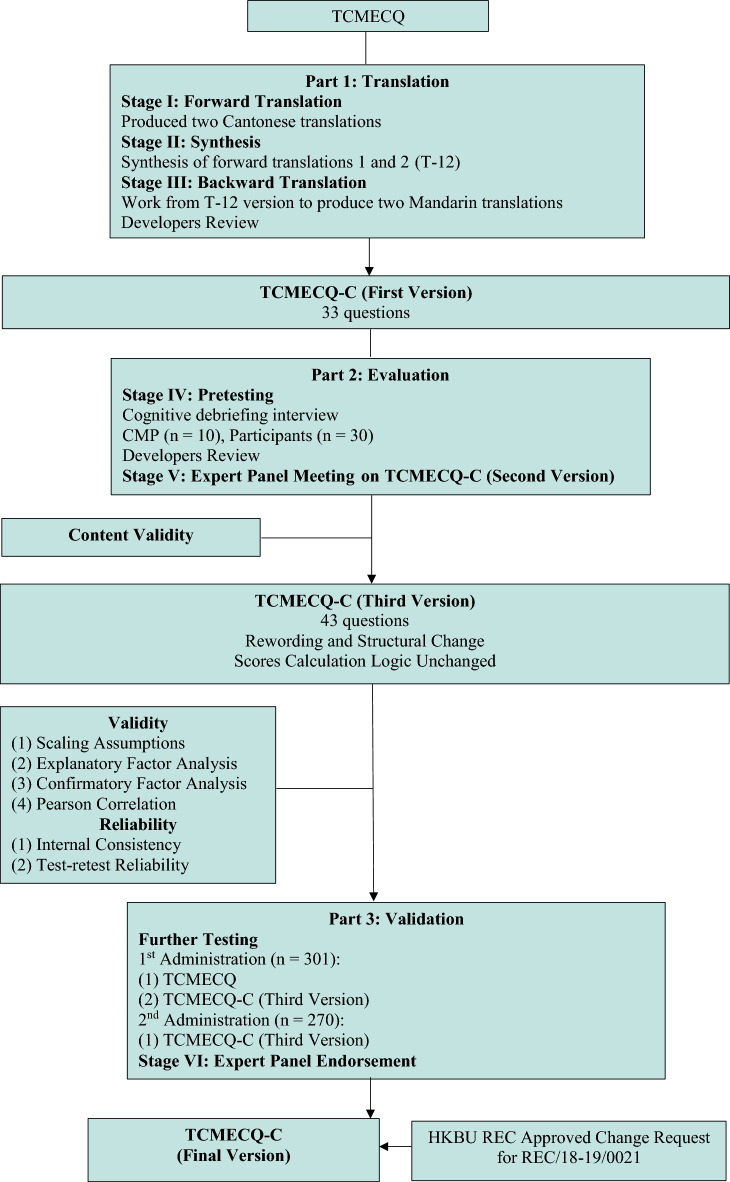
Study flowchart

This study (Fig. 1) was comprised of four parts as recommended by the International Society for Quality of Life Assessment (IQOLA) project: (1) translation of the TCMECQ (First Version); (2) Content validation based on the First Version; (3) Form the second version of TCMECQ-C based on the first validation, reviewed by the TCMECQ-C developers (ZXB and YLW) and discussed in the expert panel’s meeting, and the third version of the TCMECQ-C was then formed by the panel; (4) Construct validation of the TCMECQ-C (Third Version) [18–20].

The first phase of study was the translation of the TCMECQ into Cantonese version. Permission to translate and reword the TCMECQ-C with reference to the CCMQ was obtained from the author of the CCMQ (Cantonese version). The translation process followed the guidelines of Beaton et al.: (1) forward translation, (2) synthesis of translations, (3) back translation, (4) review by the expert committee, (5) pretesting and cognitive interviewing. Forward and backward translations were conducted in June 2019. One Chinese Medicine Practitioner (CMP) (YLW) and one social worker (SYW), both bilingual native Cantonese speakers, carried out the forward translation (Mandarin to Cantonese), while two independent bilingual translators of native Mandarin without medical background [one was a financial analyst (ZW); another was a PhD student from Communication Studies (LZ)] produced two back translations (Cantonese to Mandarin) of the questionnaire. The TCMECQ-C developers comprising the principal investigator (ZXB) and the CMP translator (YLW) produced the first version of TCMECQ-C. A volunteer group of 10 CMPs recruited from the HKBUSCM, 30 participants aged 65 and above from the community, were invited to evaluate the first version for content validity by cognitive debriefing interviews from July 2019 to August 2019 [12, 18, 19, 21, 22]. Then the TCMECQ-C developers (ZXB and YLW) reviewed the study results and produced the draft for the second version and submitted for the expert panel’s appraisal. An expert panel (see Additional file 1), including the original translator of the CCMQ, 3 experts on TCM basic theory, and 1 professional from the Faculty of Arts of HKBU, finalized the TCMECQ-C on 23 October 2019 as the Third Version [12].

In the second phase of the study, to investigate the construct validity of the TCMECQ-C (Third Version), 303 participants were recruited from December 2019 to February 2020. Each participant was assessed by a trained CMP for the participant demographic characteristics and the responses of TCMECQ, TCMECQ-C. During the first assessment, the CMP recorded the participant-provided demographics answers (e.g. gender, education) and participant’s chosen responses most reflecting their conditions. The CMP read each question of TCMECQ to the participant but did not intervene the participant’ choice for assisting in clarifying, explaining according to the original intentions of the questions to be assessed. Continuing the finished TCMECQ, without the reference to the previous answers of the responded TCMECQ, the participant answered each question of TCMECQ-C (Third Version) with also the CMP’s assistance. To evaluate test–retest reliability, the TCMECQ-C (Third Version) was again administered by the CMP to the same sample participants 2 weeks after the initial assessment [23–25]. 89.70% (270/301) participants completed the second visit for the test–retest assessment. With the accomplishment of the validation, suggested rewording of 4 questions of the TCMECQ-C was submitted to the panel of experts for discussion and finalization. Expert panel commented those questions deeply and endorsed the rewording of the 4 questions on 22 March 2020. The TCMECQ-C developers finalized the TCMECQ-C following the suggestions. In the third version of the TCMECQ-C, 16 questions had been reworded and 4 questions had been restructured into sub-questions respectively; in the validated endorsed third version (Final Version) of TCMECQ-C, 5 questions have been reworded (see Additional file 2).

### Measures

#### TCMECQ and TCMECQ-C (first version)

The TCMECQ designed for the elderly in Beijing was translated to TCMECQ-C (First Version) targeting HK elderly aged 65 and above to identify their body constitution, formulate the health regimen strategy and facilitate the elderly constitution research and disease prevention. Both the TCMECQ and TCMECQ-C (First Version) comprises 33 items (including 4 questions (item 2, 4, 5 and 13) are shared between Qi-deficiency Constitution, Yang-deficiency Constitution, Stagnant Qi Constitution and Balanced Constitution) with the items identifying the respective nine body constitutions [5, 16, 26]. All items are calculated based on the five-point Likert scale scoring algorithm [5]. All the 8 biased constitutions were the sum scores of their corresponding items, with scores ≥ 11 indicating having the assessed constitution(s). The scoring of the Balanced Constitution: item 1 + item 2 (reverse scoring) + item 4 (reverse scoring) + item 5 (reverse scoring) + item 13 (reverse scoring) ≥ 17 and with all other eight biased constitution ≤ 8.

#### TCMECQ-C (second version)

The TCMECQ-C (Second Version) comprises 52 items (including 4 questions (item 2, 4, 5 and 13) are shared between Qi-deficiency Constitution, Yang-deficiency Constitution, Stagnant Qi Constitution and Balanced Constitution) with the items identifying the respective nine body constitutions, (1) for Yang-deficiency Constitution, the original item 12 and item 27 was split into 4 and 2 sub questions respectively, making it 4 more items were added to the TCMECQ; (2) for Yin-deficiency Constitution, the original item 10, item 20 and item 29 was split into 2 sub questions respectively, making it 3 more items were added to the TCMECQ; (3) for Phlegm-dampness Constitution, the original item 22 and item 32 was split into 4 and 2 sub questions respectively, making it 4 more items were added to the TCMECQ; (4) for Dampness-heat Constitution, the original item 24 was split into 2 sub questions, making it 1 more item was added to TCMECQ; (5) for Stagnant Blood Constitution, the original item 23 was split into 2 sub questions, making it 1 more item was added to TCMECQ; (6) for Stagnant Qi Constitution, the original item 8 was split into 2 sub questions, making it 1 more item was added to TCMECQ; (7) for Inherited Special Constitution, the original item 17 was split into 6 sub questions, making it 5 more items were added to TCMECQ, [5, 16, 26] 19 more items in total were thus added to form the TCMECQ-C (Second Version). Considering some measurements were carried out for the participants by the CMP (for example, item 20 the scratch test may done by the CMP), the order of the items was rearranged (for example, item 20 was rearranged to be item 30). All items are calculated based on the five-point Likert scale scoring algorithm which was consisted with TCMECQ. All the 8 biased constitutions were the sum scores of their corresponding items, with scores ≥ 11 indicating having the assessed constitution(s). The scoring of the Balanced Constitution: item 1 + item 2 (reverse scoring) + item 4 (reverse scoring) + item 5 (reverse scoring) + item 13 (reverse scoring) ≥ 17 and with all other eight biased constitution ≤ 8. The 4 biased constitutions including Yang-deficiency Constitution, Stagnant Blood, Stagnant Qi and Inherited Special Constitutions have the consistent scoring method and the cut-off value with TCMECQ, by counting the highest score from the composed items in the corresponding subscales to get one score to be added up with other items’ scores, such as in Stagnant Qi Constitution, the score was calculated as item 5 + item 6 + item 7 + item 8 (count the highest score from “8.1, 8.2” to get one score for item 8).

#### TCMECQ-C (third version)

The TCMECQ-C (Third Version) comprises 43 items (including 4 questions are shared between Qi-deficiency Constitution, Yang-deficiency Constitution, Stagnant Qi Constitution and Balanced Constitution) with the items identifying the respective nine body constitutions, (1) for Yang-deficiency Constitution, the original item 12 was split into 4 sub questions, making it 3 more items were added to the TCMECQ; (2) for Stagnant Blood Constitution, the original item 24 was split into 2 sub questions, making it 1 more item was added to TCMECQ; (3) for Stagnant Qi Constitution, the original item 8 was split into 2 sub questions, making it 1 more item was added to TCMECQ; (4) for Inherited Special Constitution, the original item 17 was split into 6 sub questions, making it 5 more items were added to TCMECQ, [5, 16] 10 more items in total were thus added to form the TCMECQ-C (Third Version). All items are calculated based on the five-point Likert scale scoring algorithm which was consisted with TCMECQ. All the 8 biased constitutions were the sum scores of their corresponding items, with scores ≥ 11 indicating having the assessed constitution(s). The scoring of the Balanced Constitution: item 1 + item 2 (reverse scoring) + item 4 (reverse scoring) + item 5 (reverse scoring) + item 13 (reverse scoring) ≥ 17 and with all other eight biased constitution ≤ 8.

The 4 biased constitutions including Yang-deficiency Constitution, Stagnant Blood, Stagnant Qi and Inherited Special Constitutions have the consistent scoring method and the cut-off value with TCMECQ, to remain unchanged with the TCMECQ scoring method. Further, we count the highest score from the composed items in the corresponding subscales to get one score to be added up with other items’ scores, such as in Yang-deficiency Constitution, the score was calculated as item 11 + item 12 (count the highest score from “12.1, 12.2, 12.3 or 12.4” to get one score for item 12) + item 13 + item 29. The original item 12 (in Yang-deficiency Constitution), item 24 (in Stagnant Blood Constitution), item 8 (in Stagnant Qi Constitution) and item 17 (in Inherited Special Constitution) were split into sub questions respectively, aiming a more precise identification which considering important in TCM theory. For example, for Yang-deficiency Constitution, the original item 12 was split into 4 sub questions, aiming to more precisely identify the exact region of the body that afraid of cold.

### Sample size

The recommended 10 CMPs and 30 participants were tested for the content validity [18, 27]. The sample size for the designated construct validation study was determined in terms of the subject to item ratio 5:1, with reference to two review articles [28, 29]. Therefore, a sample size of at least 215 participants (5 times 43 items of the questionnaire) was needed.

### Statistical analysis

All statistical analyses were performed using SPSS (version 28) and AMOS (version 28) for Windows. Data are reported as mean $\pm$ standard deviation (SD) for continuous variables, frequency and proportion for categorical variables. Independent sample t-test and Pearson correlation were used with the normal distribution of the data [30]. Two-sided p < 0.05 was considered significant.

Content validity index (CVI) (Evaluation Study Sample) examined content domain to ensure the item covered the intended TCMECQ-C (First Version) [30, 31]. Item-level content validity indices (I-CVIs) were calculated by dividing the number of CMPs and the participants that rated grade 3 (quite relevant/clear/appropriate) or 4 (highly relevant/clear/appropriate) on the four-point Likert scale of the first three questions in the cognitive debriefing table by the total number of assessments of the interview respectively [31–33]. An item-level content validity index (I-CVI) ≥ 0.78 was considered acceptable for at least nine experts [27, 31, 34].

#### Validity (validation study sample)

Scaling assumptions was used to determine whether the item scores of each scale can be summated, were tested by the summated rating method, including (1) item frequency distribution (to ascertain whether all of the response choices were used), (2) equivalence of item means and standard deviations (i.e. approximately equivalent within a scale, to check the floor and ceiling effects) [35]. Ceiling and floor effects were defined usually with ≥ 15% of participants in a sample achieving the best/maximum or the worst/minimum score, disabling the measure to discriminate between subjects at either extreme of the scale [36]. A critical ratio > 3 with p < 0.05 (calculated by independent sample t-test) indicating an item had good discriminability [16]. Explanatory Factor Analysis (EFA) was used to confirmed the dimensionality [37, 38]. Three major steps included a) assessment of the suitability of the data (recommended sample size i.e. ten cases for each item the study sample size (n = 270) met the requirement for factor analysis [37, 38]. Coefficient of correlation > 0.30 between the variables evidenced the suitable strength of the relationship among the items. Determinant score > 0.00001. Kaiser–Meyer–Olkin > 0.60 and Bartlett’s test of Sphericity < 0.05) [37, 38]; b) factor extraction (9 factors (according to the known original 9-constitution scale structure) were extracted using principal component analysis (PCA). Considering the TCM theory, the items were specifically selected indicating a factor and comprising an essential part of the factor, the item(s) were included even if they did not reach the suggested factor loading level [39]. The required cumulative variance percentage was ≥ 50% [37]. and c) factor rotation interpretation using orthogonal factor rotation approach with the related varimax method developed by Kaiser (1958), to minimize the significant cross loadings (i.e. many factors were correlated with many variables) usually obtained in the initial extraction phase. The factor rotation method solved the interpretation of the items loaded factors (i.e. avoided the number of items having high loadings on each factor). The diagonal anti-image correlation value of each item must be > 0.5 to inform the sampling adequacy. The factor loading > 0.30 indicating that the items represent the underlying factors [37, 40, 41].

Confirmatory Factor Analysis (CFA) model fit was performed on the overall scale and subscales using AMOS. The model fit of the overall scale excluding four items in the Balanced Constitution subscale due to the reverse scores of Q2, Q4 (both shared with Qi-deficiency Constitution), Q5 (shared with Stagnant Qi Constitution) and Q13 (shared with Yang-deficiency Constitution). Statistical parameters evaluated in the CFA process including (1) the absolute fitting statistics: goodness-of-fit index (GFI), adjusted goodness-of-fit index (AGFI), root mean square error of approximation (RMSEA) and standardized root mean square residual (SRMR); (2) the incremental fitting statistics: normed fit index (NFI), incremental fit index (IFI), Tucker–Lewis index (TLI), comparative fit index (CFI) and relative fit index (RFI); and (3) the parsimony fitting statistics: the chi-squared degree-of-freedom ratio (χ2/DF). GFI, AGFI, NFI, IFI, TLI, CFI and RFI ≥ 0.90, RMSEA and SRMR ≤ 0.08 and χ2/DF between 1.00 and 3.00, implied excellent CFA model fit [16]. Pearson correlation, i.e. Pearson correlation coefficient (r) was investigated for the construct validity (convergent validity and divergent validity) among the nine subscales of TCMECQ-C [35, 42]. Sufficient convergent validity between the subscales were represented by (1) moderate to strong correlation: 0.30 < r < 0.70, (2) strong correlation: r ≥ 0.70; sufficient divergent validity between the subscales: r ≤ 0.30) [35]. Assuming moderate to strong correlations among the three constitutions (Qi-deficiency Constitution, Yang-deficiency Constitution and Yin-deficiency Constitution) as they are measuring the deficiency construct and other five constitutions (Phlegm-dampness Constitution, Dampness-heat Constitution, Stagnant Blood Constitution, Stagnant Qi Constitution, Inherited Special Constitution) as they are measuring the excessive construct. Assuming low correlations between deficiency subscales and excessive subscales as the hypotheses for the divergent validation.

#### Reliability (validation study sample)

Internal consistency was measured by Cronbach’s alpha [43]. A rule-of-thumb good scale consistency was defined with the Cronbach’s alpha coefficient between 0.50 and 0.70 based on the scale dimensionality [44]. Hinton et al. suggested four cut-off points for a more concrete reliability acceptable level including excellent reliability (0.90 and above), high reliability (0.70–0.90), moderate reliability (0.50–0.70) and low reliability (0.50 and below), was adopted to reveal the true reliability of the scale [45]. A lower Cronbach’s alpha was considered sufficient in indicating consistency for scales with less than 10 items [12, 44–46]. The corrected item-total correlation defined the association of the item with the total score on the other items [44, 47, 48]. Corrected item-total correlation using a correlation coefficient ≥ 0.40 as the cut-off for adequate correlation [45, 49]. Moreover, a correlation higher than 0.20 suggested that each item has a good correlation with the scale [44]. Items having item-to-total correlation less than 0.20 were retained if Cronbach’s alpha did not increase upon deleting these items [44, 47, 48]. Test–retest reliability was evaluated by Intra-class correlation coefficients (ICC) [50, 51], in which using the single-measurement (comparing TCMECQ-C (Third Version) with TCMECQ), absolute-agreement, the two-way mixed effects model, the ICC results based on the 95% confident interval of the ICC estimate were then reported [51]. Values < 0.50 were indicative of poor reliability, values between 0.5 and 0.75 indicated moderate reliability, values between 0.75 and 0.9 indicated good reliability, and values > 0.90 indicated excellent reliability [51].

## Results

### Demographic characteristics

All 10 CMPs were academically qualified with at least the Bachelor of TCM degrees and 80.00% (8/10) of them have experience in clinical research of TCM. The CMPs have an average of 10.60 (SD = 4.58) years of clinical experience (see Additional file 3). The 30 participants for evaluation study were aged 65–80 years old and 56.67% (17/30) of them were Secondary School-educated (see Additional file 3). 48.15% (130/270) participants for validation study were Secondary School-educated (see Additional file 4).

### Translation validity

All items of the TCMECQ-C (First Version) were calculated to be ranging from 0.80 to 1.00 on clarity, relevance and appropriateness by the CMPs and the participants, except the items of Phlegm-dampness Constitution (Q16 and Q28), Stagnant Blood Constitution (Q19 and Q22), Stagnant Blood Constitution (Q5–Q8), Inherited Special Constitution (Q20) and Balanced Constitution (Q5) rated low relevance by the participants (Table 1).

**Table 1:** Item-level content validity index of TCMECQ-C (First Version) rated by the CMPs and the participants

Item	Clarity		Relevance		Appropriateness	
	CMPs (n = 10)	Participants (n = 30)	CMPs (n = 10)	Participants (n = 30)	CMPs (n = 10)	Participants (n = 30)
BC 1						
2	1.00	1.00	1.00	0.93	0.90	1.00
3	1.00	1.00	1.00	0.93	0.90	1.00
4	1.00	1.00	1.00	0.80	0.90	1.00
14	1.00	1.00	1.00	1.00	0.90	0.97
BC 2						
11	1.00	1.00	1.00	0.97	0.90	1.00
12	1.00	1.00	1.00	1.00	0.90	0.97
13	1.00	1.00	1.00	1.00	0.90	1.00
29	1.00	1.00	1.00	0.97	0.90	0.93
BC 3						
10	0.90	1.00	1.00	0.90	0.90	1.00
21	1.00	1.00	1.00	0.93	0.90	1.00
26	1.00	1.00	1.00	0.93	0.90	1.00
31	1.00	1.00	1.00	0.97	0.90	1.00
BC 4						
9	0.90	0.90	1.00	0.87	0.90	0.97
16	1.00	1.00	0.90	0.70	0.90	1.00
28	1.00	0.93	1.00	0.77	0.50	0.67
32	1.00	0.93	1.00	0.97	0.90	0.93
BC 5						
23	1.00	1.00	1.00	0.87	0.90	1.00
25	1.00	1.00	1.00	0.97	0.90	1.00
27	1.00	1.00	1.00	0.93	0.90	1.00
30	1.00	0.97	1.00	0.93	0.90	0.97
BC 6						
19	1.00	0.97	1.00	0.73	0.90	0.97
22	1.00	0.87	0.80	0.60	0.90	0.97
24	1.00	0.97	1.00	0.90	0.80	0.97
33	1.00	0.90	1.00	0.87	0.80	0.90
BC 7						
5	1.00	1.00	1.00	0.53	0.90	1.00
6	1.00	1.00	1.00	0.60	0.90	1.00
7	1.00	0.97	1.00	0.53	0.90	1.00
8	1.00	1.00	1.00	0.57	0.90	1.00
BC 8						
15	1.00	1.00	1.00	1.00	0.90	1.00
17	1.00	0.97	1.00	0.80	0.90	0.93
18	1.00	0.87	1.00	0.83	0.90	1.00
20	0.90	1.00	0.80	0.70	0.90	1.00
BC 9						
1	1.00	1.00	1.00	0.93	0.90	1.00
2^a^	1.00	1.00	1.00	0.93	0.90	1.00
4^a^	1.00	1.00	1.00	0.80	0.90	1.00
5^a^	1.00	1.00	1.00	0.53	0.90	1.00
13^a^	1.00	1.00	1.00	1.00	0.90	1.00

BC1: Qi-deficiency Constitution, BC2: Yang-deficiency Constitution, BC3: Yin-deficiency Constitution, BC4: Phlegm-dampness Constitution, BC5: Dampness-heat Constitution, BC6: Stagnant Blood Constitution, BC7: Stagnant Qi Constitution, BC8: Inherited Special Constitution, BC9: Balanced Constitution

An I-CVI ≥ 0.78 was considered acceptable for at least nine experts

^a^Item with reverse scoring: (1) “No” reversed to “All the time”, (2) “Slightly” reversed to “Often”, (3) “Sometimes” remained “Sometimes”, (4) “Often” reversed to “Slightly”, (5) “All the time” reversed to “No”

### Validity

#### Scaling assumption

All the response choices on the nine subscales were used (Table 2). The means and SD within the corresponding hypothesized subscales were roughly equivalent. No ceiling effect was detected (Table 3). The critic ratio value of the overall scale calculated by the independent-sample t test > 3 (t = 15.55) with significant difference (p < 0.001) (Table 4) [52].

**Table 2:** Item frequency distribution percentage of TCMECQ-C (Third Version)

Item	No (1)	Slightly (2)	Sometimes (3)	Often (4)	All the time (5)
BC 1					
2	10.37	33.33	41.85	12.22	2.22
3	45.93	32.59	15.93	4.81	0.74
4	70.00	15.56	8.15	5.93	0.37
14	46.30	41.85	7.78	2.96	1.11
BC 2					
11	50.00	12.59	21.11	12.59	3.70
12.1	82.22	5.19	7.41	4.44	0.74
12.2	61.11	5.93	20.00	11.11	1.85
12.3	74.07	6.30	12.22	6.30	1.11
12.4	61.85	7.41	19.30	9.63	1.85
12^b^	49.30	7.41	25.19	15.19	2.96
13	40.74	16.67	20.37	17.78	4.44
29	65.93	11.48	8.15	10.74	3.70
BC 3					
10	81.85	7.41	6.30	3.70	0.74
21	20.74	21.85	35.56	17.41	4.44
26	29.30	17.41	30.00	17.04	6.30
31	50.00	24.07	18.15	6.67	1.11
BC 4					
9	48.15	17.78	19.63	12.59	1.85
16	52.59	21.85	14.07	9.63	1.85
28	24.07	26.30	20.00	27.04	2.59
32	38.52	30.37	20.37	7.04	3.70
BC 5					
23	46.30	21.11	17.04	13.70	1.85
25	76.67	12.59	8.89	1.48	0.37
27	38.89	17.04	29.63	10.74	3.70
30	47.78	15.56	25.93	8.15	2.59
BC 6					
19	81.48	7.78	8.52	2.22	0.00
22	90.37	4.07	3.33	1.48	0.74
24.1	63.70	14.44	9.63	10.37	1.85
24.2	27.41	37.04	19.63	12.59	3.33
24^b^	24.44	34.44	19.30	18.15	3.70
33	38.52	31.85	17.04	10.00	2.59
BC 7					
5	43.70	25.19	26.30	4.81	0.00
6	32.59	30.74	28.89	7.41	0.37
7	52.59	25.56	17.78	4.07	0.00
8.1	57.04	27.04	11.48	4.44	0.00
8.2	48.52	28.15	15.93	7.41	0.00
8^b^	45.93	30.37	15.93	7.78	0.00
BC 8					
15	44.44	19.30	20.74	13.33	2.22
17.1	88.52	5.19	4.07	0.74	1.48
17.2	85.19	7.04	5.19	1.48	1.11
17.3	80.00	7.04	8.15	3.33	1.48
17.4	89.63	3.33	4.81	1.85	0.37
17.5	71.85	7.41	14.44	5.19	1.11
17.6	75.19	8.15	10.37	5.56	0.74
17^b^	55.19	10.74	20.00	10.37	3.70
18	87.41	5.19	5.19	2.22	87.41
20	78.15	10.00	7.78	3.33	0.74
BC 9					
1	0.74	7.41	34.44	46.67	10.74
2^a^	10.37	33.33	41.85	12.22	2.22
4^a^	70.00	15.56	8.15	5.93	0.37
5^a^	43.70	25.19	26.30	4.81	0.00
13^a^	40.74	16.67	20.37	17.78	4.44

BC 1: Qi-deficiency Constitution, BC 2: Yang-deficiency Constitution, BC 3: Yin-deficiency Constitution, BC 4: Phlegm-dampness Constitution, BC 5: Dampness-heat Constitution, BC 6: Stagnant Blood Constitution, BC 7: Stagnant Qi Constitution, BC 8: Inherited Special Constitution, BC 9: Balanced Constitution

No Missing data. Data are presented as %

^a^Item with reverse scoring: (1) “No” reversed to “All the time”, (2) “Slightly” reversed to “Often”, (3) “Sometimes” remained “Sometimes”, (4) “Often” reversed to “Slightly”, (5) “All the time” reversed to “No”

^b^Items counted with the highest score: Item 8 (count the highest score from 8.1 or 8.2), Item 12 (count the highest score from 12.1, 12.2. 12.3 or 12.4), Item 17 (count the highest score from 17.1, 17.2. 17.3, 17.4, 17.5 or 17.6) and Item 24 (count the highest score from 24.1 or 24.2)

**Table 3:** Mean, SD, floor and ceiling effects of TCMECQ-C (Third Version)

Item	Mean	SD	Floor effect (%)	Ceiling effect (%)
BC 1				
2	2.63	0.91	10.37	2.22
3	1.82	0.92	45.93	0.74
4	1.51	0.90	70.00	0.37
14	1.71	0.82	46.30	1.11
BC 2				
11	2.07	1.24	50.00	3.70
12.1	1.36	0.86	82.22	0.74
12.2	1.87	1.19	61.11	1.85
12.3	1.54	1.00	74.07	1.11
12.4	1.82	1.16	61.85	1.85
12^b^	2.15	1.27	49.30	2.96
13	2.29	1.28	40.74	4.44
29	1.75	1.20	65.93	3.70
BC 3				
10	1.34	0.82	81.85	0.74
21	2.63	1.13	20.74	4.44
26	2.54	1.25	29.30	6.30
31	1.85	1.01	50.00	1.11
BC 4				
9	2.02	1.16	48.15	1.85
16	1.86	1.10	52.59	1.85
28	2.58	1.20	24.07	2.59
32	2.07	1.10	38.52	3.70
BC 5				
23	2.04	1.16	46.30	1.85
25	1.36	0.74	76.67	0.37
27	2.23	1.18	38.89	3.70
30	2.02	1.14	47.78	2.59
BC 6				
19	1.31	0.72	81.48	0.00
22	1.18	0.63	90.37	0.74
24.1	1.72	1.12	63.70	1.85
24.2	2.27	1.10	27.41	3.33
24^b^	2.42	1.15	24.44	3.70
33	2.06	1.09	38.52	2.59
BC 7				
5	2.12	0.97	43.70	0.00
6	1.73	0.89	32.59	0.37
7	1.63	0.86	52.59	0.00
8.1	1.82	0.96	57.04	0.00
8.2	2.12	0.97	48.52	0.00
8^b^	1.86	0.96	45.93	0.00
BC 8				
15	2.10	1.18	44.44	2.22
17.1	1.21	0.69	88.52	1.48
17.2	1.26	0.72	85.19	1.11
17.3	1.39	0.88	80.00	1.48
17.4	1.20	0.64	89.63	0.37
17.5	1.56	0.99	71.85	1.11
17.6	1.49	0.94	75.19	0.74
17^b^	1.97	1.23	55.19	3.70
18	1.22	0.64	87.41	87.41
20	1.39	0.83	78.15	0.74
BC 9				
1	3.59	0.81	0.74	10.74
2^a^	3.37	0.91	10.37	2.22
4^a^	4.49	0.90	70.00	0.37
5^a^	4.08	0.94	43.70	0.00
13^a^	3.71	1.28	40.74	4.44

BC 1: Qi-deficiency Constitution, BC 2: Yang-deficiency Constitution, BC 3: Yin-deficiency Constitution, BC 4: Phlegm-dampness Constitution, BC 5: Dampness-heat Constitution, BC 6: Stagnant Blood Constitution, BC 7: Stagnant Qi Constitution, BC 8: Inherited Special Constitution, BC 9: Balanced Constitution

No Missing data. Data are presented as %

^a^Item with reverse scoring: (1) “No” reversed to “All the time”, (2) “Slightly” reversed to “Often”, (3) “Sometimes” remained “Sometimes”, (4) “Often” reversed to “Slightly”, (5) “All the time” reversed to “No”

^b^Items counted with the highest score: Item 8 (count the highest score from 8.1 or 8.2), Item12 (count the highest score from 12.1, 12.2. 12.3 or 12.4), Item 17 (count the highest score from 17.1, 17.2. 17.3, 17.4, 17.5 or 17.6) and Item 24 (count the highest score from 24.1 or 24.2)

**Table 4:** Critical ratio of the overall scale of TCMECQ-C (Third Version)

Mean		Levene’s test		t	Two-sided p
Low score	High score	F	p^*^		
150.72 (± 61.14)	5.14 (± 5.43)	87.61	< 0.001	15.55	< 0.001

Data are presented as mean ± standard deviation

^*^Sig. value p > 0.05, equal variances assumed

#### Explanatory factor analysis

The determinant score was 0.00003077 indicating that an absence of multicollinearity in the data, which made it be suitable for factor analysis [53]. The Kaiser–Meyer–Olkin value (0.84) indicated that the adequate sampling; the approximate chi-square of the Bartlett’s test of Sphericity of the total scale was 2671.69 with the degrees of freedom ratio 528 (p < 0.001) showed that the data set was worth for a factor analysis [37].

After extracting the nine factors, each eigenvalue of the factors was > 1 along with the cumulative variance percentage of the total variance was 57.60%, shared by 33 variables could be accounted by nine factors.

The diagonal anti-image correlation value of each item ranging from 0.53 to 0.93 testified the sampling adequacy. The factor loading for Qi-deficiency Constitution (factor 1) ranging from 0.37 to 0.71; Yang-deficiency Constitution (factor 4) ranging from 0.36 to 0.65; Yin-deficiency Constitution (factor 5) ranging from 0.36 to 0.65; Phlegm-dampness Constitution (factor 6) Q16 = 0.84 except Q9, Q28 and Q32; Dampness-heat Constitution (factor 3) ranging from 0.44 to 0.73 except Q23 and Q25; Stagnant Blood Constitution (factor 9) was 0.72 except Q19, Q24 (the highest score) and Q33, Stagnant Qi Constitution (factor 2) ranging from 0.68 to 0.82 and Inherited Special Constitution (factor 7) ranging from 0.63 to 0.71 except for Q18 and Q20, met the required factor loading (> 0.30); excluding Balanced Constitution had three positive factor loadings (one shared with factor 6, another shared with factor 7 and the third one shared with factor 9) (Table 5) [41].

**Table 5:** Factor analysis on TCMECQ-C (Third Version) items

Item	Factor loading								
	1	2	3	4	5	6	7	8	9
BC1									
2	**0.71**	0.19	0.16	0.06	− 0.02	0.03	0.14	0.10	0.02
3	**0.70**	0.24	− 0.08	0.03	0.15	− 0.07	0.09	− 0.09	− 0.04
4	**0.42**	0.29	− 0.08	**0.43**	0.20	− 0.13	0.16	− 0.11	− 0.19
14	**0.37**	0.04	0.21	0.10	0.21	0.10	**0.35**	− 0.19	− **0.42**
BC2									
11	0.20	0.13	0.16	**0.65**	0.12	− 0.05	0.01	0.07	0.19
12^a^	**0.35**	0.15	0.28	**0.43**	− 0.01	0.09	0.23	0.19	0.20
13	**0.33**	**0.31**	0.17	**0.45**	− 0.03	0.05	0.21	0.07	0.00
29	0.24	0.11	0.29	**0.36**	− 0.13	0.09	0.25	0.28	− 0.05
BC3									
10	0.23	− 0.13	0.03	0.05	**0.39**	**0.33**	0.20	0.01	0.00
21	0.05	0.19	0.19	0.08	**0.55**	0.00	0.22	0.12	0.12
26	0.14	0.17	**0.41**	0.09	**0.36**	− 0.20	**0.34**	0.01	− 0.10
31	0.11	0.15	0.09	0.07	**0.65**	− 0.03	− 0.08	0.19	0.08
BC4									
9	**0.65**	0.24	0.29	− 0.15	0.15	0.09	− 0.01	− 0.02	0.09
16	− 0.02	0.11	0.01	0.01	− 0.03	**0.84**	0.07	0.06	− 0.01
28	0.29	− 0.19	0.07	− **0.72**	− 0.11	0.11	0.03	0.03	0.08
32	0.14	− 0.03	**0.70**	0.07	0.12	0.14	0.00	0.02	− 0.02
BC5									
23	0.04	0.08	0.07	− 0.09	0.08	**0.79**	0.07	− 0.06	0.17
25	− 0.08	0.13	0.09	0.02	**0.42**	**0.34**	0.04	0.07	− 0.25
27	0.11	0.19	**0.73**	0.01	0.00	0.05	0.24	0.02	− 0.01
30	0.15	0.23	**0.44**	0.06	0.19	− 0.18	0.13	0.18	0.13
BC6									
19	**0.33**	0.16	0.12	0.14	**0.35**	0.12	− 0.12	0.24	0.08
22	− 0.01	− 0.05	0.04	0.07	0.13	0.17	0.25	− 0.06	**0.72**
24^a^	0.11	0.23	**0.42**	0.24	0.30	− 0.08	− 0.14	− 0.09	0.30
33	0.06	− 0.01	**0.45**	**0.38**	**0.31**	0.21	− 0.08	− 0.04	− 0.09
BC7									
5	0.23	**0.78**	0.13	0.03	0.17	0.14	0.04	− 0.04	− 0.08
6	0.16	**0.82**	0.07	0.15	0.08	0.02	0.08	0.05	0.08
7	**0.31**	**0.77**	0.07	0.12	0.11	0.02	0.04	0.05	− 0.03
8^a^	0.16	**0.68**	0.13	**0.34**	0.08	0.09	0.07	0.05	− 0.05
BC8									
15	0.01	0.00	0.09	− 0.04	0.15	0.14	**0.71**	− 0.07	0.07
17^a^	0.12	0.19	0.08	0.18	− 0.10	0.03	**0.63**	0.18	0.13
18	− 0.02	− 0.05	− 0.16	0.03	0.25	− 0.08	0.10	**0.76**	0.08
20	0.05	0.09	0.20	0.03	0.09	0.07	− 0.04	**0.75**	− 0.10
BC9									
1	− 0.72	− 0.15	− 0.18	− 0.17	− 0.03	0.00	0.04	− 0.10	0.05

Extraction Method: Principal Component Analysis

Rotation Method: Varimax with Kaiser Normalization

Factor loadings > 0.3 are given bold

BC1: Qi-deficiency Constitution, BC2: Yang-deficiency Constitution, BC3: Yin-deficiency Constitution, BC4: Phlegm-dampness Constitution, BC5: Dampness-heat Constitution, BC6: Stagnant Blood Constitution, BC7: Stagnant Qi Constitution, BC8: Inherited Special Constitution, BC9: Balanced Constitution

^a^Items counted with the highest score: Item 8 (count the highest score from 8.1 or 8.2), Item 12 (count the highest score from 12.1, 12.2. 12.3 or 12.4), Item 17 (count the highest score from 17.1, 17.2. 17.3, 17.4, 17.5 or 17.6) and Item 24 (count the highest score from 24.1 or 24.2)

#### Confirmatory factor analysis (CFA)

**Fig. 2:**
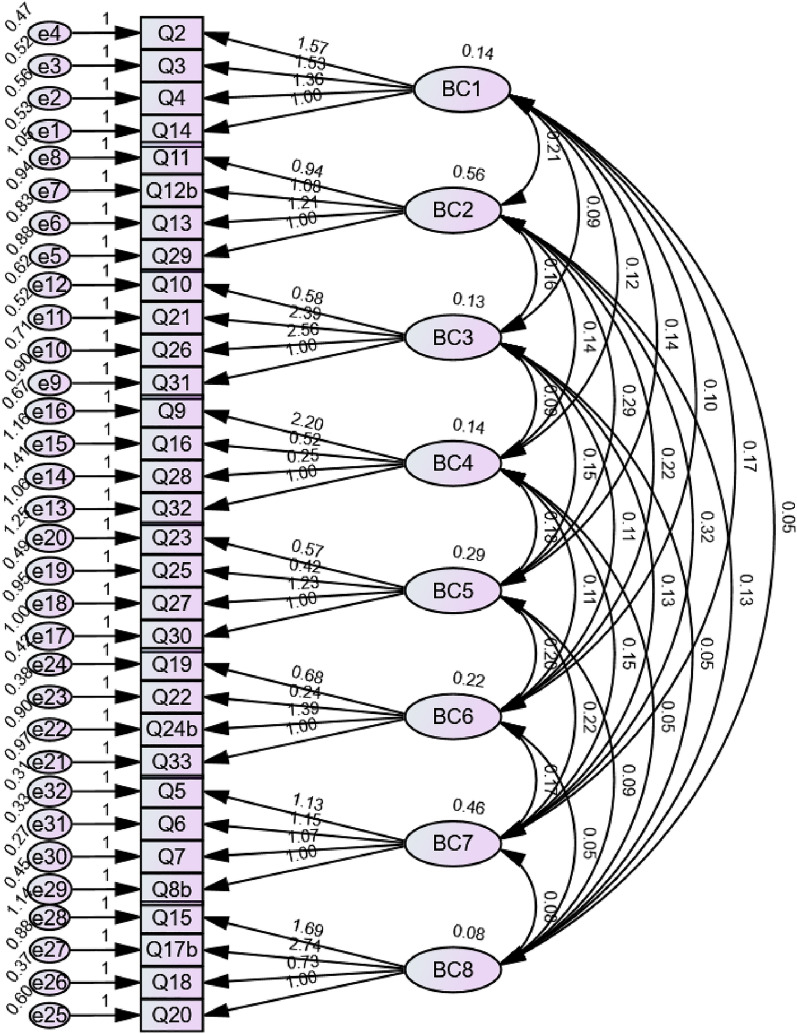
Model fit for the overall scale of TCMECQ-C (Third Version) (excluding Balanced Constitution). BC 1: Qi-deficiency Constitution, BC 2: Yang-deficiency Constitution, BC 3: Yin-deficiency Constitution, BC 4: Phlegm-dampness Constitution, BC 5: Dampness-heat Constitution, BC 6: Stagnant Blood Constitution, BC 7: Stagnant Qi Constitution, BC 8: Inherited Special Constitution. ^b^Items counted with the highest score: Item 8 (count the highest score from 8.1 or 8.2), Item 12 (count the highest score from 12.1, 12.2. 12.3 or 12.4), Item 17 (count the highest score from 17.1, 17.2. 17.3, 17.4, 17.5 or 17.6) and Item 24 (count the highest score from 24.1 or 24.2)

**Fig. 3:**
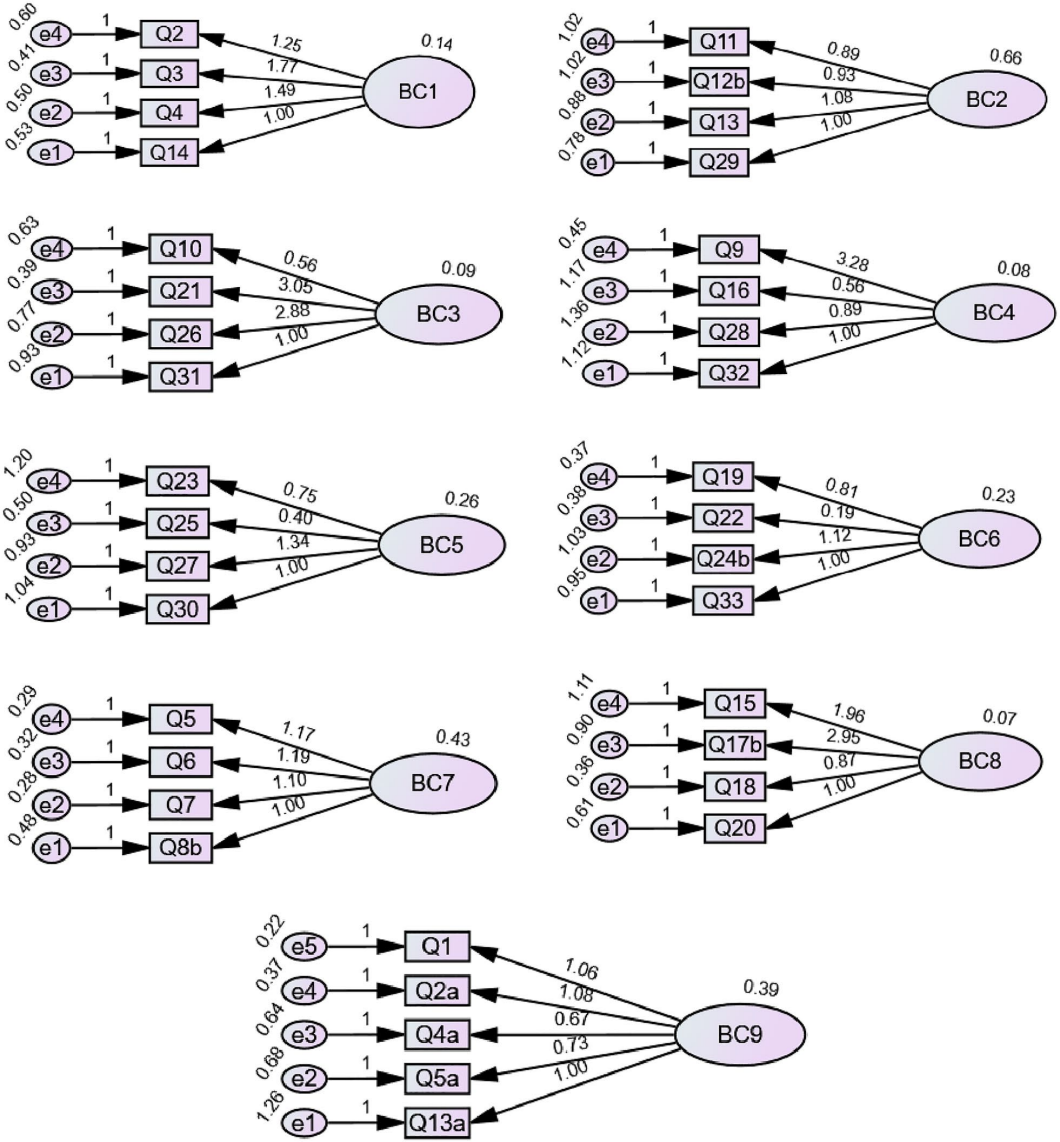
Model fit for the subscales of TCMECQ-C (Third Version). BC 1: Qi-deficiency Constitution, BC 2: Yang-deficiency Constitution, BC 3: Yin-deficiency Constitution, BC 4: Phlegm-dampness Constitution, BC 5: Dampness-heat Constitution, BC 6: Stagnant Blood Constitution, BC 7: Stagnant Qi Constitution, BC 8: Inherited Special Constitution, BC 9: Balanced Constitution. ^a^Item with reverse scoring: (1) “No” reversed to “All the time”, (2) “Slightly” reversed to “Often”, (3) “Sometimes” remained “Sometimes”, (4) “Often” reversed to “Slightly”, (5) “All the time” reversed to “No”. ^b^Items counted with the highest score: Item 8 (count the highest score from 8.1 or 8.2), Item12 (count the highest score from 12.1, 12.2. 12.3 or 12.4), Item 17 (count the highest score from 17.1, 17.2. 17.3, 17.4, 17.5 or 17.6) and Item 24 (count the highest score from 24.1 or 24.2)

The model fits of the overall scale (excluding Balanced Constitution) (Fig. 2) and the nine subscales (Fig. 3) were acceptable. The χ2/DF of the overall scale was 2.13 (928.63/436), indicating a satisfactory model fit. The subscales model fitting results: GFI was between 0.95 and 1.00 and AGFI was 0.85 and 0.99 (0.91 in Qi-deficiency Constitution, 0.99 in Yang-deficiency Constitution, 0.97 in Yin-deficiency Constitution, 0.98 in Phlegm-dampness Constitution, 0.87 in Dampness-heat Constitution, 0.99 in Stagnant Blood Constitution, 0.86 in Stagnant Qi Constitution, 0.89 in Inherited Special Constitution and 0.85 in Balanced Constitution), which were considered acceptable. NFI was between 0.74 and 0.99, CFI was between 0.75 and 1.00, and SRMR was between 0.01 and 0.06, which were all considered acceptable. IFI was between 0.77 and 1.01, RFI was between 0.23 and 0.98, TLI was between 0.25 and 1.04, and RMSEA was between 0.00 and 0.16 (Table 6).

**Table 6:** Model fit results for the overall scale and the subscales of TCMECQ-C (Third Version)

Index	Overall scale	CFA model fitting index of subscale								
		BC 1	BC 2	BC 3	BC 4	BC 5	BC 6	BC 7	BC 8	BC 9
GFI	0.83	**0.98**	**1.00**	**0.99**	**1.00**	**0.97**	**1.00**	**0.97**	**0.98**	**0.95**
AGFI	0.79	**0.91**	**0.99**	**0.97**	**0.98**	0.87	**0.99**	0.86	0.89	0.85
NFI	0.66	**0.94**	**0.99**	**0.97**	**0.90**	0.74	**0.97**	**0.97**	0.81	**0.90**
CFI	0.78	**0.95**	**1.00**	**0.99**	**0.97**	0.75	**1.00**	**0.97**	0.83	**0.91**
SRMR	**0.07**	**0.04**	**0.01**	**0.03**	**0.03**	**0.06**	**0.02**	**0.03**	**0.06**	**0.06**
IFI	0.78	**0.95**	**1.00**	**0.99**	**0.97**	0.77	**1.01**	**0.97**	0.84	**0.91**
RFI	0.61	0.81	**0.98**	**0.92**	0.71	0.23	**0.90**	**0.90**	0.44	0.79
TLI	0.75	0.84	**1.01**	**0.96**	**0.90**	0.25	**1.04**	**0.91**	0.49	0.82
RMSEA	**0.07**	0.12	**0.00**	**0.06**	**0.04**	0.15	**0.00**	0.16	0.14	0.14
χ2/DF	**2.13**	5.01	**0.82**	**1.88**	**1.38**	7.15	0.77	8.19	5.91	6.36

BC 1: Qi-deficiency Constitution, BC 2: Yang-deficiency Constitution, BC 3: Yin-deficiency Constitution, BC 4: Phlegm-dampness Constitution, BC 5: Dampness-heat Constitution, BC 6: Stagnant Blood Constitution, BC 7: Stagnant Qi Constitution, BC 8: Inherited Special Constitution, BC 9: Balanced Constitution

*CFA* Confirmatory Factor Analysis, *GFI* Goodness-of-Fit Index, *AGFI* Adjusted Goodness-of-Fit Index, *NFI* Normed Fit Index, *CFI* Comparative Fit Index, *SRMR* Standardized Root Mean Square Residual, *IFI* Incremental Fit Index, *RFI* Relative Fit Index, *TLI* Tucker–Lewis Index, *RMSEA* Root Mean Square Error of Approximation, *χ2/DF* Chi-squared Degree-of-Freedom Ratio

Indexes values of GFI, AGFI, NFI, RFI, IFI, TLI, and CFI ≥ 0.90 indicate ideal or acceptable model fitting. Indexes values of SRMR or RMSEA ≤ 0.08 indicate ideal or acceptable model fitting. The value between 1.00 and 3.00 is needed for χ2/DF to indicate a good model fitting, or else it is not satisfactory

The indexes values indicated an ideal or acceptable or a good model fitting are given bold

## Correlation

Data of 270 participants who completed the TCMECQ-C (Third Version) at the first administration were analyzed. Phlegm-dampness Constitution and Dampness-heat Constitution had moderate to strong correlation (r = 0.35, p < 0.01) established the expected sufficient convergent validity. Yang-deficiency Constitution established the sufficient divergent validity with the 5 excessive body constitution including Dampness-heat Constitution (r = 0.22, p < 0.01) etc. Yin-deficiency Constitution established the sufficient divergent validity with all the 5 excessive body constitutions including Phlegm-dampness Constitution (r = 0.22, p < 0.01) etc. Worth attention, Balanced Constitution was negatively correlated with all other eight constitution subscales, suggesting it measured body constitution from a reverse perspective comparing to that of the other constitutions (Table 7).

**Table 7:** Correlations among the subscale scores of TCMECQ-C (Third Version)

Subscale	BC 1	BC 2	BC 3	BC 4	BC 5	BC 6	BC 7	BC 8	BC 9
BC 1	1								
BC 2	0.29^**^	1							
BC 3	0.21^**^	0.28^**^	1						
BC 4	0.08^b^	0.11^b^	0.22^**b^	1					
BC 5	0.07^b^	0.22^**b^	0.15^*b^	0.35^**a^	1				
BC 6	0.13^*b^	0.20^**b^	0.21^**b^	0.14^*^	0.18^**^	1			
BC 7	0.37^**^	0.28^**b^	0.29^**b^	0.09	0.018^**^	0.18^**^	1		
BC 8	0.11^b^	0.30^**b^	0.18^**b^	0.16^*^	0.16^**^	0.11	0.13^*^	1	
BC 9	− 0.19^**^	− 0.29^**^	− 0.26^**^	− 0.26^**^	− 0.21^**^	− 0.14^*^	− 0.23^**^	− 0.15^*^	1

BC 1: Qi-deficiency Constitution, BC 2: Yang-deficiency Constitution, BC 3: Yin-deficiency Constitution, BC 4: Phlegm-dampness Constitution, BC 5: Dampness-heat Constitution, BC 6: Stagnant Blood Constitution, BC 7: Stagnant Qi Constitution, BC 8: Inherited Special Constitution, BC 9: Balanced Constitution

^**^Correlation is significant at the 0.01 level (2-tailed)

^*^Correlation is significant at the 0.05 level (2-tailed)

^a^Sufficient convergent validity:

(1) Moderate to strong correlation: 0.30 < r < 0.70

(2) Strong correlation: r ≥ 0.70

^b^Sufficient of divergent validity between the scales: r ≤ 0.30

### Reliability

#### Internal consistency

A good scale consistency of the seven body constitutions were demonstrated with the Cronbach’s alpha between 0.56 and 0.89. The values for Phlegm-dampness Constitution (0.34) and Dampness-heat Constitution (0.44) in TCMECQ-C (Third Version) suggested the low reliability, were considered sufficient indicating consistency for scales with 4 items [44] (Table 8). The Cronbach’s alpha will increase from (1) 0.59 to 0.63 when deleting Q10 (corrected item-total correlation 0.21), (2) 0.56 to 0.60 when deleting Q22 (corrected item-total correlation 0.06) and (3) 0.76 to 0.77 when deleting Q15 (corrected item-total correlation 0.32) [47]. Q22 with corrected item-total correlation < 0.20, when deleted will only increase the Cronbach’s alpha slightly, may considered to be retained.

#### Test–retest stability

The baseline and after 2-week ($\pm$ 3 days) data (n = 270) was assessed. The level of significance for ICC was set at P < 0.05. The ICC of the nine body constitutions of TCMECQ-C ranged from 0.70 to 0.87 (p < 0.001), indicating moderate to good test–retest reliability of the subscale scores [51] (Table 8).

**Table 8:** Internal consistency and ICC of TCMECQ-C (Third Version)

Item	Corrected item-total correlation coefficient	Cronbach's alpha coefficient if item deleted
BC 1 (Cronbach’s alpha coefficient: 0.67) [Intra-class correlation coefficient^c*^: 0.75^b^ (0.69, 0.81)]		
2	0.43	0.62
3	0.52	0.55
4	0.46	0.59
14	0.39	0.64
BC 2 (Cronbach’s alpha coefficient: 0.84) [Intra-class correlation coefficient^c*^: 0.81^b^ (0.76, 0.85)]		
11	0.46	0.84
12.1	0.56	0.82
12.2	0.70	0.80
12.3	0.69	0.80
12.4	0.63	0.81
13	0.60	0.81
29	0.54	0.82
BC 3 (Cronbach’s alpha coefficient: 0.59) [Intra-class correlation coefficient^c*^: 0.74^b^ (0.68, 0.79)]		
10	0.21	0.63
21	0.54	0.38
26	0.49	0.42
31	0.28	0.59
BC 4 (Cronbach’s alpha coefficient: 0.34) [Intra-class correlation coefficient^c*^: 0.79^b^ (0.74, 0.84)]		
9	0.28	0.15
16	0.14	0.31
28	0.13	0.33
32	0.16	0.29
BC 5 (Cronbach’s alpha coefficient: 0.44) [Intra-class correlation coefficient^c*^: 0.73^b^ (0.67, 0.78)]		
23	0.23	0.39
25	0.23	0.40
27	0.31	0.30
30	0.24	0.38
BC 6 (Cronbach’s alpha coefficient: 0.56) [Intra-class correlation coefficient^c*^: 0.87^b^ (0.84, 0.90)]		
19	0.32	0.51
22	0.06	0.60
24.1	0.50	0.37
24.2	0.38	0.46
33	0.33	0.50
BC 7 (Cronbach’s alpha coefficient: 0.89) [Intra-class correlation coefficient^c*^: 0.82^b^ (0.76, 0.86)]		
5	0.71	0.87
6	0.74	0.86
7	0.73	0.86
8.1	0.75	0.86
8.2	0.72	0.87
BC 8 (Cronbach’s alpha coefficient: 0.76) [Intra-class correlation coefficient^c*^: 0.70^b^ (0.63, 0.76)]		
15	0.32	0.77
17.1	0.39	0.74
17.2	0.42	0.74
17.3	0.57	0.71
17.4	0.58	0.72
17.5	0.67	0.69
17.6	0.57	0.71
18	0.25	0.76
20	0.24	0.76
BC 9 (Cronbach’s alpha coefficient: 0.73) [Intra-class correlation coefficient^c*^: 0.84^b^ (0.80, 0.88)]		
1	0.59	0.65
2^a^	0.52	0.67
4^a^	0.45	0.69
5^a^	0.47	0.69
13^a^	0.46	0.71

BC 1: Qi-deficiency Constitution, BC 2: Yang-deficiency Constitution, BC 3: Yin-deficiency Constitution, BC 4: Phlegm-dampness Constitution, BC 5: Dampness-heat Constitution, BC 6: Stagnant Blood Constitution, BC 7: Stagnant Qi Constitution, BC 8: Inherited Special Constitution, BC 9: Balanced Constitution

^*^p < 0.001

^a^Item with reverse scoring: (1) “No” reversed to “All the time”, (2) “Slightly” reversed to “Often”, (3) “Sometimes” remained “Sometimes”, (4) “Often” reversed to “Slightly”, (5) “All the time” reversed to “No”

^b^The estimator is the same, whether the interaction effect is present or not

^c^Type A intra-class correlation coefficients using an absolute agreement definition

## Discussion

The I-CVI of all items of the TCMECQ-C (First Version) were found acceptable on clarity by the CMPs and the participants. For the TCMECQ-C (Third Version), no ceiling effect was observed; factor analysis showed 4 constructs were identified consistent with TCMECQ-C (Third Version); CFA model fit results supported the validity of the respective overall scale and subscales constructs; Pearson’s r showed the sufficient convergent validity and sufficient divergent validity for excessive subscales; seven body constitutions had a good Cronbach alpha; ICC analysis showed moderate to good test–retest reliability of all the subscale scores.

The floor effect was identified in most of the questions, which can occur in resiliency measures (TCMECQ-C measures elderly endurance and adaptation throughout the life process from time to time) [54–57]. The other 4 corresponding constructed subscales (excluding Balanced Constitution) were not extracted via EFA as same as the expected hypothesized 9 body constitutions. This may due to an inappropriate construct of the measurement or the misunderstanding of the questions leading to the non-representative responses of the participants [39]. Considering the acceptable results of CFA, the EFA was overcome for the reasons: (1) founded on the TCM theoretical basis, the results can be explained and comprehended. For instance, Phlegm-dampness (cold in nature) is usually formed as the deficiency in Qi/Yang Qi to transform/warm the circulating body fluid but condense the fluid to the cold dampness, vice versa when the cold dampness (an evil of Yin) defeated Yang Qi/hindered Qi to warm/transform the circulating body fluid, the sluggish fluid is then appeared as phlegm and dampness. This gives the possibility for the Phlegm-dampness Constitution shared the same factors with Qi-deficiency Constitution and Yang-deficiency Constitution. Dampness-heat Constitution shared the same factors with Yin-deficiency Constitution and Phlegm-dampness Constitution. As deficiency in Yin may give rise to thickening the body fluid, the thickened fluid became difficult to flow. If the stagnant fluid stayed too long in the body, it would get heated up to damp heat. The values of the respective four items composing the Yin-deficiency Constitution and Dampness-heat Constitution were similar as shown in Fig. 2, explaining their overlapping in the same factor. Internal dampness combines with either cold or heat leading to damp heat or damp cold and the interchangeability of the two statuses may explain their co-existence in the same factor. Stagnant Blood Constitution appeared in the same factors with Qi-deficiency Constitution, Yang-deficiency Constitution and Yin-deficiency Constitution matching the TCM theory. The deficiency in Qi, Yang and Yin (the vital energy) to motivate the blood circulation causes the stasis of blood. The blood (hot in nature/fire element) is also one of the body fluid components closely related to the heat and vice versa, may account for their sharing in the common factor. Inherited Special Constitution was found in factor 7 and factor 8, independent from other factors with its special nature more prone to arise from inheritance and allergy. (2) currently there is no clear rule indicating when an item with a factor loading that is too low to be included or excluded, but assume the items were specifically selected in indicating a factor and some items are comprising an essential part of the factor when they are theoretically distant from the factor resulting a low factor loading, the research team may not easily conclude the item(s) were not belong to their scale(s) [39]. (3) CFA is considered more suitable to confirm the number of common factors in a structured questionnaire (the best models is “9” for the TCMECQ-C based on the TCM Constitutional Theory) [58]. Some subscales with less than 10 items had a relative low Cronbach’s alpha coefficient especially for Phlegm-dampness Constitution and Dampness-heat Constitution, which may due to an insufficient quantities of questions, unsatisfactory inter-relatedness between items and/or heterogeneous constructs [46]. As Cronbach alpha measures the reliability or consistency between several items, high alpha values can be achieved given enough item, thus higher of alpha value(s) is/are not necessarily better because it could indicate lengthy scales, item redundancy or construct’s narrow coverage or construct underrepresentation etc. A low Cronbach’s alpha value can be yielded because of the inadequate numbers of items etc [45]. The results of Cronbach’s alpha coefficient suggested that more fit-to-scale questions shall be constructed for the Phlegm-dampness Constitution and Dampness-heat Constitution to promote a more acceptable internal consistency.

Furthermore, though guidelines were followed [18], limitations during the translation, evaluation and validation process should be noted. For translation process, usually linguistics professional, methodologist etc. shall be involved [18]. The study with its confined resources, social worker, financial analyst and postgraduate student of Communication supported the study. For evaluation study, the content validity of the TCMECQ-C (First Version) was rated by the CMPs and the participants but not the face validity of the experts. The experts were engaged to have an in-depth discussion in revising the constructs of the questions and the scoring algorithm. For validation study, a larger sample size is better to attain a more representative factor analysis etc.

As far as we know, there was not yet any similar works done by other researchers, while in 2013, Wong W. et al. proved the CCMQ (Cantonese version) to be valid and reliable for the HK Chinese population, the study’s targeted population was aged ≥ 18 years old (Cognitive debriefing patients (n = 10): aged 18–44 years old (n = 5), aged 45–64 years old (n = 3) and > 65 (n = 2); Patients (n = 1,084): age 48.9 ± 14.8) [12]. The consistent questions of the TCMECQ-C with the CCMQ (Cantonese version) were reworded with reference to Wong et al.’s translation for the Cantonese-speaking elderly. The questionnaire did not develop directly from Wendy’s version to Elderly Cantonese Version (TCMECQ-C) for CCMQ was originally developed to target population aged 16 to 65 years old [8] or 18 to 60 years old [5]. With the availability of TCMECQ, it is a usual practice to develop its corresponding Cantonese version aiming to use the specified measurement identifying the HK elderly’s body constitution and most importantly through the correct use of the instrument, a following preventive strategy will then be formulated appropriately.

## Conclusion

The TCMECQ-C is shown to be as reliable and valid as the TCMECQ, is thus validated as a convenient self-assessed TCM questionnaire for the differentiation of body constitutions of the HK elderly population, optimizing clinical prevention therapy for existing disease states.

The results of this study were expected to benefit other researchers and TCM practitioners in HK and around the world serving Cantonese (Yueyu)-speaking elderly populations. It provided valuable reference for developing health status survey measures in other Asian populations, such as Singapore.

### Supplementary Information

**Additional file 1: **Name of panel experts.pdf. The 5 experts who participated in the panel meeting to produce the TCMECQ-C (Third Version) and endorsed the 4 rewording questions to produce the TCMECQ-C (Final Version).

**Additional file 2: **The differences between the “First Version”, “Third Version” and “Final Version” of TCMECQ-C.pdf. The comparison of TCMECQ-C (Third Version) to the First Version, 16 questions were reworded, and 4 questions were restructured into sub-questions respectively; The comparison of TCMECQ (Final Version) to the Third Version, 5 questions were reworded.

**Additional file 3: **Demographic characteristics of the CMPs and the participants for the evaluation study.pdf. Ten CMPs and the 30 participants’ demographic characteristics were reported.

**Additional file 4: **Demographic characteristics of the participants for the validation study.pdf. Two hundred and seventy participants’ demographic characteristics were reported.

## Data Availability

All relevant data are included in this manuscript. The datasets used and/or analyzed during the current study are available from the corresponding author on reasonable request.

## Abbreviations

TCM: Traditional Chinese Medicine
TCMECQ: The TCM Body Constitution Questionnaire (For Elderly People)
HK: Hong Kong
CMPs: Chinese Medicine Practitioners
TCMECQ-C: Traditional Chinese Medicine Body Constitution Questionnaire (For Elderly People) (Cantonese version)
r: Pearson correlation coefficient
CCMQ: Constitution in Chinese Medicine Questionnaire
REC: Research Ethics Committee
HKBU: Hong Kong Baptist University
SCM: School of Chinese Medicine
IQOLA: International Society for Quality of Life Assessment
SD: Standard deviation
CVI: Content validity index
I-CVIs: Item-level content validity indices
EFA: Explanatory Factor Analysis
CFA: Confirmatory Factor Analysis
GFI: Goodness-of-fit index
AGFI: Adjusted goodness-of-fit index
RMSEA: Root mean square error of approximation
SRMR: Standardized root mean square residual
NFI: Normed fit index
IFI: Incremental fit index
TLI: Tucker–Lewis index
CFI: Comparative fit index
RFI: Relative fit index
χ2/DF: Chi-squared degree-of-freedom ratio
ICC: Intra-class correlation coefficients
