# Supplementary material for: Validation of the Cantonese version of the Traditional Chinese Medicine (TCM) Body constitution Questionnaire in elderly people
Source: Chin Med. 2023 Oct 11;18:129. doi: 10.1186/s13020-023-00805-w (PMC10566140; doi:10.1186/s13020-023-00805-w)
Supplement: Supplementary file 1 — Additional file 1: Name of panel experts.pdf. The 5 experts who participated in the panel meeting to produce the TCMECQ-C (Third Version) and endorsed the 4 rewording questions to produce the TCMECQ-C (Final Version). [file 13020_2023_805_MOESM1_ESM.pdf]

Additional file 1. Name of panel experts

No.	Name
1.	Dr Hon Man Chan
2.	Dr Wendy Wong
3.	Dr Qing Ling Zhang
4.	Dr Lan O
5.	Dr Lijuan Yu

Note: Expert 1: professional from the Faculty of Arts of HKBU, Expert 2: original translator of the CCMQ, Expert 3-5: experts on TCM basic theory. Names not listed in order.
