# Supplementary material for: Validation of the Cantonese version of the Traditional Chinese Medicine (TCM) Body constitution Questionnaire in elderly people
Source: Chin Med. 2023 Oct 11;18:129. doi: 10.1186/s13020-023-00805-w (PMC10566140; doi:10.1186/s13020-023-00805-w)
Supplement: Supplementary file 2 — Additional file 2: The differences between the “First Version”, “Third Version” and “Final Version” of TCMECQ-C.pdf. The comparison of TCMECQ-C (Third Version) to the First Version, 16 questions were reworded, and 4 questions were restructured into sub-questions respectively; The comparison of TCMECQ (Final Version) to the Third Version, 5 questions were reworded. [file 13020_2023_805_MOESM2_ESM.pdf]

Additional file 2. The differences between the "First Version", "Third Version" and "Final Version" of TCMECQ-C

Item	TCMECQ-C (First Version)	Item	TCMECQ-C (Third Version) First Version vs Third Version	Item	TCMECQ-C (Final version) Third Version vs Final Version
Q4	您說話聲音低、弱及無力嗎？ Did you speak in a low, weak and lack of strength voice?	Q4	您說話聲音低弱無力嗎？ Did you feel feeble when talking?	N/A	N/A
Q7	您多愁善感、情緒脆弱嗎？ Did you sentimental, emotionally fragile?	Q7	您多愁善感、感情脆弱嗎？ Did you sentimental, sensationally fragile?	N/A	N/A
Q8	您容易覺得害怕或容易受到驚嚇嗎？ Did you easily get scared or being frightened?	Q8.1	您容易覺得害怕嗎？ Did you easily get scared?	N/A	N/A
		Q8.2	您容易受到驚嚇嗎？ Did you easily be frightened?	N/A	N/A
Q9	您覺得身體沉重不爽快嗎？	Q9	您感到身體沉重不輕鬆嗎？	Q9	您感到身體困重不輕鬆嗎？

Q11	Did your body feel heavy and uncomfortable?<br>您手腳冰涼嗎？<br>Did your extremities feel cold?	Q11	Did your body feel heavy and uneasy?<br>您手腳冰冷嗎？<br>Did your extremities feel chilled?	N/A	Did your body feel damp and uneasy?<br>N/A
Q12	您胃部、背部、腰部或膝部怕冷嗎？<br>Did your stomach, back, lumber or knee region afraid of cold?	Q12.1	您上腹部怕冷嗎？<br>Did your upper abdomen region afraid of cold?	N/A	N/A
		Q12.2	您背部怕冷嗎？<br>Did your back region afraid of cold?	N/A	N/A
		Q12.3	您腰部怕冷嗎？<br>Did your lumber region afraid of cold?	N/A	N/A
		Q12.4	您膝關節怕冷嗎？<br>Did your knee region afraid of cold?	N/A	N/A
Q16	您額頭油脂分泌多嗎？	Q16	您前額油脂分泌多嗎？	N/A	N/A

	Did your forehead have excessive excretion?		Did your forehead (upper third of the face in between the hairline and the eyebrows) have excessive excretion?		
Q17	您容易過敏嗎（對藥物、食物、氣味、花粉或在季節交替、氣候變化時）？ Did you easily get allergic (to the medications, the food, the smell, pollens or during the seasonal change, climate change)?	Q17.1	您對藥物容易過敏嗎？ Did you allergic to the medications easily?	Q17.1	您對藥物過敏嗎？ Did you allergic to the medications?
		Q17.2	您對食物容易過敏嗎？ Did you allergic to the food easily?	Q17.2	您對食物過敏嗎？ Did you allergic to the food?
		Q17.3	您對氣味容易過敏嗎？ Did you allergic to the smell easily?	Q17.3	您對氣味過敏嗎？ Did you allergic to the smell?
		Q17.4	您對花粉容易過敏嗎？ Did you allergic to the pollens easily?	Q17.4	您對花粉過敏嗎？ Did you allergic to the pollens?
		Q17.5	您在季節交替時容易過敏嗎？	N/A	N/A

		Q17.6	Did you get allergic easily during the seasonal change?<br>您在氣候變化時容易過敏嗎？<br>Did you get allergic easily during the climate change?	N/A	N/A
Q18	您的皮膚容易起蕁麻疹（風團、風疹塊、風疙瘩）嗎？<br>Did your skin easily have Urticaria (wheal, raised lumps, skin swelling)?	Q18	您的皮膚容易起風疹（包括風團、風疹塊、風疙瘩）嗎？<br>Did your skin easily have Acute Urticaria (wheal, raised lumps, skin swelling)?	N/A	N/A
Q22	您的兩顴有細微紅絲嗎？<br>Did your cheek have small capillaries?	N/A	N/A	Q22	您面頰上顴骨部位看得到微絲血管嗎？<br>Did you see the capillaries on your zygomatic area of your face?

Q23	您的面或鼻會油膩或油亮發光嗎？<br>Did your face or nose get greasy or oily?	Q23	您的面部或鼻部有油膩感嗎？<br>Did your face area or nose area has a greasy feeling?	N/A	N/A
Q24	您面色暗沉無光澤或容易出現黃褐斑嗎？<br>Did your face have a dull complexion or easily have chloasma?	Q24.1	您面色暗淡嗎？<br>Did your face have a gloomy complexion?	N/A	N/A
		Q24.2	您面部容易出現老人斑嗎？<br>Did your face easily have age spots?	N/A	N/A
Q25	您面部容易生暗瘡或皮膚容易生膿瘡嗎？<br>Did your face easily have acnes or abscesses?	Q25	您易生粉刺、暗瘡或膿瘡嗎？<br>Did your easily have comedo, acnes or abscesses?	N/A	N/A
Q27	您覺得口苦嗎？<br>Did your mouth feel bitter?	Q27	您覺得口苦或口腔有異味嗎？<br>Did your mouth feel bitter or smells?	N/A	N/A

Q28	您腹部肥大嗎？<br><br>Did you have a large belly?	N/A	N/A	Q28	您腹部肥大嗎？(站立位, 以肚臍為中線用皮尺平臍部繞腹部測量腹圍)<br><br>Did you have a large belly (standing position, tape measuring the abdominal circumstance with the umbilicus as the median)?
Q29	您吃（喝）冰涼的東西會感到不舒服或者不想吃（喝）冰涼東西嗎？<br><br>Did you feel uncomfortable when eating (drinking) the cold food or did not want to eat (drink) the cold food?	Q29	您食/喝凍的食物/飲品會感到不舒服或者怕食/喝凍的食物/飲品嗎？<br><br>Did you feel uncomfortable when eating / drinking the frozen food / drink or afraid to eat / drink the frozen food / drink?	N/A	N/A

Q30	您大便黏滯不爽快、有去不清的感覺嗎？<br>Did your stool sluggish with an uncomfortable, unclear bowel movement feeling?	Q30	您的大便黏糊不爽、有未排清的感覺嗎？<br>Did your stool sticky with an uncomfortable, undischarged bowel movement feeling?	N/A	N/A
Q31	您容易便秘或大便質地乾燥嗎？<br>Did you easily have constipation or your stool dry in texture?	Q31	您容易便秘或大便乾燥嗎？<br>Did you easily have constipation or your stool was dry?	N/A	N/A
Q33	您舌下靜脈瘀紫嗎?<br>Did your sublingual vein bruise?	N/A	N/A	Q33	您舌底下的靜脈是暗紫色嗎？<br>Were your sublingual vein (underneath the tongue) dark purple in colour?

Note: English translations with reference to CCMQ-EE by Song, H., et al., *Revision and validation of the "Constitution in Chinese Medicine Questionnaire (Elderly Edition)" based on the Delphi process.* Journal of Traditional Chinese Medical Sciences, 2022. 9(3): p. 246-256.
