# Supplementary material for: Validation of the Cantonese version of the Traditional Chinese Medicine (TCM) Body constitution Questionnaire in elderly people
Source: Chin Med. 2023 Oct 11;18:129. doi: 10.1186/s13020-023-00805-w (PMC10566140; doi:10.1186/s13020-023-00805-w)
Supplement: Supplementary file 3 — Additional file 3: Demographic characteristics of the CMPs and the participants for the evaluation study.pdf. Ten CMPs and the 30 participants’ demographic characteristics were reported. [file 13020_2023_805_MOESM3_ESM.docx]

**Additional file 3.** Demographic characteristics of the CMPs and the participants for the evaluation study

| **Characteristics** | **CMPs (n=10)** | **Participants (n = 30)** |
| --- | --- | --- |
| **Age** (Year, mean ± SD) | 37.44 ± 3.61 | 69.77 ± 3.59 |
| **Gender (n (%))** | | |
| Male | N/A | 7 (23.30) |
| Female | N/A | 23 (76.70) |
| **Marital Status (n (%))** | | |
| Unmarried | N/A | 4 (13.30) |
| Married | N/A | 16 (53.30) |
| Separated/Divorced | N/A | 4 (13.30) |
| Widow/Widower | N/A | 6 (20.00) |
| **Education (n (%))** | | |
| Bachelor | 1 (10.00) | N/A |
| MPhil | 1 (10.00) | N/A |
| PhD | 8 (80.00) | N/A |
| No Formal Schooling | N/A | 1 (3.30) |
| Kindergarten/ Primary School | N/A | 3 (10.00) |
| Secondary School (Form1-Form 3) | N/A | 6 (20.00) |
| Secondary School (Form4-Form 5) | N/A | 11 (36.70) |
| Form6-7/ Hong Kong Institute of Vocational Education (IVE/VTC) | N/A | 2 (6.70) |
| Associate Degree | N/A | 3 (10.00) |
| University or above | N/A | 4 (13.30) |
| **Clinical Experience** **(Overall)** (Years, mean ± SD): 15.50 ± 9.85 | | N/A |
| **Clinical Experience (in HK)** (Years, mean ± SD): 10.60 ± 4.58 | | N/A |
| **Clinical Research Experience** (n (%)) | | |
| Yes | 8 (80.00) | N/A |
| No | 2 (20.00) | N/A |
| **Occupation (n (%))** | | |
| Manager and administrators | N/A | 3 (10.00) |
| Professionals | N/A | 6 (20.00) |
| Clerks | N/A | 5 (16.70) |
| Service workers and shop sales workers | N/A | 4 (13.30) |
| Self-employed | N/A | 4 (13.30) |
| Housewife | N/A | 5 (16.70) |
| Others | N/A | 3 (10.00) |

Note: Data are presented as mean ± standard deviation or number (%).
