# Supplementary material for: Validation of the Cantonese version of the Traditional Chinese Medicine (TCM) Body constitution Questionnaire in elderly people
Source: Chin Med. 2023 Oct 11;18:129. doi: 10.1186/s13020-023-00805-w (PMC10566140; doi:10.1186/s13020-023-00805-w)
Supplement: Supplementary file 4 — Additional file 4: Demographic characteristics of the participants for the validation study.pdf. Two hundred and seventy participants’ demographic characteristics were reported. [file 13020_2023_805_MOESM4_ESM.docx]

**Additional file 4.** Demographic characteristics of the participants for the validation study

| Characteristics | Participants (n = 270) |
| --- | --- |
| Age (Year, mean ± SD): 70.03 ± 3.56 | |
| Gender (n (%)) | |
| Male | 92 (34.07) |
| Female | 178 (65.93) |
| Education (n (%)) | |
| Kindergarten | 10 (3.70) |
| Primary School | 59 (21.85) |
| Secondary School (Form1-Form 3) | 62 (22.96) |
| Secondary School (Form4-Form 5) | 68 (25.19) |
| Form6-7/ Hong Kong Institute of Vocational Education (IVE/VTC) | 24 (8.89) |
| Associate Degree | 17 (6.30) |
| University or above | 30 (11.11) |
| Occupation (n (%)) | |
| Manager and administrators | 25 (9.26) |
| Professionals | 34 (12.59) |
| Associate professionals | 5 (1.85) |
| Clerks | 49 (18.15) |
| Service workers and shop sales workers | 47 (17.41) |
| Craft and related workers | 27 (10.00) |
| Machine operators and assemblers | 10 (3.70) |
| Unskilled workers | 13 (4.81) |
| Farm workers, animal husbandry workers and fishermen, and occupations unidentifiable and inadequately described | 2 (0.74) |
| Self-employed | 12 (4.44) |
| Housewife | 24 (8.89) |
| Others | 22 (8.15) |
| Marital Status (n (%)) | |
| Unmarried | 31 (11.48) |
| Married | 192 (71.11) |
| Widow/Widower | 25 (9.26) |
| Separated/Divorced | 22 (8.15) |
| Abdominal Circumstance (cm, mean ± SD): 86.24 ± 9.70 | |
| Abdominal Circumstance (Chinese Foot, mean ± SD): 2.59 ± 0.29 | |

Note: Data are presented as mean ± standard deviation or number (%).
